# Evaluating the Coverage and Potential of Imputing the Exome Microarray with Next-Generation Imputation Using the 1000 Genomes Project

**DOI:** 10.1371/journal.pone.0106681

**Published:** 2014-09-09

**Authors:** Erwin Tantoso, Lai-Ping Wong, Bowen Li, Woei-Yuh Saw, Wenting Xu, Peter Little, Rick Twee-Hee Ong, Yik-Ying Teo

**Affiliations:** 1 Saw Swee Hock School of Public Health, National University of Singapore, Singapore, Singapore; 2 Life Sciences Institute, National University of Singapore, Singapore, Singapore; 3 Department of Statistics and Applied Probability, National University of Singapore, Singapore, Singapore; 4 NUS Graduate School for Integrative Science and Engineering, National University of Singapore, Singapore, Singapore; 5 Genome Institute of Singapore, Agency for Science, Technology and Research, Singapore, Singapore; University of Alabama at Birmingham, United States of America

## Abstract

Next-generation genotyping microarrays have been designed with insights from large-scale sequencing of exomes and whole genomes. The exome genotyping arrays promise to query the functional regions of the human genome at a fraction of the sequencing cost, thus allowing large number of samples to be genotyped. However, two pertinent questions exist: firstly, how representative is the content of the exome chip for populations not involved in the design of the chip; secondly, can the content of the exome chip be imputed with the reference data from the 1000 Genomes Project (1KGP). By deep whole-genome sequencing two Asian populations that are not part of the 1KGP, comprising 96 Southeast Asian Malays and 36 South Asian Indians for which the same samples have also been genotyped on both the Illumina 2.5 M and exome microarrays, we discovered the exome chip is a poor representation of exonic content in our two populations. However, up to 94.1% of the variants on the exome chip that are polymorphic in our populations can be confidently imputed with existing non-exome-centric microarrays using the 1KGP panel. The coverage further increases if there exists population-specific reference data from whole-genome sequencing. There is thus limited gain in using the exome chip for populations not involved in the microarray design. Instead, for the same cost of genotyping 2,000 samples on the exome chip, performing whole-genome sequencing of at least 35 samples in that population to complement the 1KGP may yield a higher coverage of the exonic content from imputation instead.

## Introduction

Genome-wide association studies (GWAS) have successfully identified thousands of single nucleotide polymorphisms (SNPs) that are associated with common diseases and complex traits [1]. The attention of these studies has primarily been directed at common variants, defined as possessing a minor allele frequency of at least 5%, by virtue of the design of GWAS that leveraged on linkage disequilibrium and the detection of surrogate associations at tagging SNPs [2], [3]. Disappointingly, the discoveries from GWAS have only accounted for a fraction of the phenotypic variance for the majority of the outcomes [4], [5]. The search for factors accounting for the missing heritability has thus shifted to interrogate functional regions of the human genome, such as the gene exons, with the hope to locate low-frequency or rare variants that contribute a greater impact to disease biology [6]–[8].

While exome sequencing provides an unbiased survey of exonic variants, the cost is still prohibitive to hundreds or thousands of samples for an association study. Next-generation genotyping microarrays designed with information from population-level whole-genome and whole-exome sequencing provide a well-intentioned compromise for surveying the genome with a genotyping approach at a fraction of the cost. For example, the Illumina HumanExome array included coding variants that were consistently identified across multiple individuals from a collection of around 12,000 subjects from diverse populations with African, Chinese, European and Hispanic ancestries, and this microarray was competitively priced with the intention of extending its use to existing GWAS cohorts. However, one of the challenges of working with exonic variants at the rarer end of the allele frequency spectrum is the greater tendency for these variants to be population specific [9], [10]. This raises the question whether an exome-centric array that is designed with prior information from a subset of populations will be relevant for other populations of different ancestries.

Statistical imputation has been widely employed in GWAS for inferring the genotypes at SNPs that are not available on the microarrays but are present in population-level haplotype reference data [11], such as those from the International HapMap Project [12], [13] or the 1000 Genomes Project (1KGP) [10], [14]. Imputation methods such as IMPUTE [15], BEAGLE [16] or MACH [17] rely on patterns of genetic correlation in the reference maps in order to identify the likely genotype for each sample at an untyped SNP. While the availability of a cosmopolitan reference panel from 1KGP is expected to improve imputation accuracy [18], [19], whether this is similarly true for exonic variants may depend on multiple factors, such as the ancestry of the target population, the choice of the reference panel, and the allele frequency spectrum of the exonic variants.

Here, we aim to address the following questions: (i) to what extent does the Illumina Exome chip represent the exonic content of two Asian populations that are not involved in the microarray design? (ii) for studies where there are pre-existing GWAS data, to what extent can the exonic content of these populations be recovered accurately through the process of statistical imputation against the reference map from Phase 1 of the 1KGP, thereby removing the need for additional genotyping on the exome chip? (iii) does having population-specific haplotype maps on top of the 1KGP reference maps help in recovering the exonic variants that are present in these populations? To answer these questions, we utilized the resources from: (a) the Singapore Integrative Omics Study (iOmics), where each of 110 southern Han Chinese, 108 Southeast Asian Malays and 105 south Asian Indians in Singapore has been genotyped on both the Illumina HumanOmni2.5 and Illumina HumanExome microarrays; (b) the Singapore Sequencing Study, where 96 Singapore Malays and 36 Singapore Indians from the iOmics have been whole-genome sequenced to a target coverage of 30-fold; and (c) the haplotype reference maps from Phase 1 of the 1KGP. By down-sampling the variants on the HumanOmni2.5, we recreated the SNP content on earlier generations of microarrays such as the HumanHap550 and Human1M for evaluating how well these commonly-used products can recover the content of the exome chip.

## Materials and Methods

### Sample collection

The Singapore Integrative Omics Study (iOmics) surveyed 120 individuals from each of three populations in Singapore consisting of southern Han Chinese, Southeast Asian Malays and south Asian Indians. These individuals were recruited from the Singapore Population Health Study, and population membership of each subject was determined through self-reports that all four grandparents belong to the same population. Each of the 360 subjects was genotyped on both the Illumina HumanOmni2.5 and HumanExome microarrays. A subset of these samples were additionally whole-genome sequenced to a target coverage of 30-fold. These subset of samples include 62 of the Malays and 36 of the Indians. Note that subsequent assessment of imputation coverage and accuracy was restricted to the subset of Malay and Indian subjects that have not been sequenced in order to avoid over-fitting. All participants provided written informed consent, and ethical approvals for the Singapore Population Health Study and the subsequent extension to the iOmics were granted from two independent Institutional Review Boards at the National University Hospital Singapore and the National University of Singapore respectively. In particular, the consent form was approved by the Institutional Review Boards prior to the commencement of the sample recruitment. The data for both the microarray genotyping and whole-genome sequencing can be accessed publicly at http://www.statgen.nus.edu.sg.

### Microarray genotyping data

Quality control (QC) of the two sets of genetic data (Omni2.5, exome chip) was performed independently and in the following three steps: (A) a preliminary QC step performed across all 360 samples jointly to generate a set of pseudo-clean SNPs by removing SNPs (i) with either unknown or duplicate genomic coordinates, (ii) not in the autosomal and X/Y chromosomes, (iii) with unknown strand information, (iv) with conflicting allele designation between the two microarrays, (v) that occur on both microarrays but with genotype concordance <99.5%, (vi) with missingness >5%, or (vii) with gross departure from Hardy-Weinberg equilibrium (HWE p-value <10^−8^); (B) a sample QC step performed across all 360 samples jointly to identify problematic samples, defined as samples (i) with high missingness (>2%), (ii) with excessive identify-by-state (IBS) genotypes (see **Table S1**), or (iii) that were genetically inferred to be outliers with principal components analysis (PCA) from the self-reported population membership; (C) a final round of SNP QC performed for each population to exclude SNPs with more than 5% missing genotypes or with HWE p-value <0.001. This yields a final set of 110 Chinese, 108 Malay and 105 Indian subjects with post-QC data for both the Omni2.5 and exome chip, with a similar number of SNPs that passed QC in the three populations (see **Table S2**).

For calculating IBS between samples as well as identifying outliers, one more filtering step is performed to remove SNPs with MAF <5%. IBS calculation was performed using PLINK v1.09 [28]. PLINK provides the function to estimate the genomewide IBS/IBD-sharing coefficients between the individuals from whole-genome data. Using these metrics, we could potentially identify undetected relationships between samples. For excessive IBS sharing between samples, the sample with higher call rate is retained.

For identifying outliers using PCA, all genotypes are represented in 0, 1 and 2 and missing data is −1. Data is centered and the covariance matrix is constructed. Subsequently, the projection vectors (Principal Components) are obtained from the covariance matrix. We run PCA on two set of data, i.e. with the HapMap data which consists of 1396 individuals from different populations and with only the three Singapore population groups. Samples that displayed evidence of admixture or discordance between self-reported and genetically inferred population membership are identified and excluded from the study.

### Building the Illumina HumanHap550 and Human1M

As the iOmics samples have only been genotyped on the Omni2.5 and exome chip, subsets of the SNPs on Omni2.5 were taken to mimic the content of the Illumina HumanHap550 and Illumina Human1M microarrays. Given that only 377,563 SNPs and 681,328 SNPs on the Omni2.5 overlapped with the contents of the HumanHap550 and Human1M respectively, we searched for surrogates that existed on the Omni2.5 to recover the effective coverage of the latter two microarrays. This is achieved by considering the CEU and JPT+CHB resource from the 1000 Genomes Project, where to identify a surrogate for a target SNP, we consider the following situations in a hierarchical fashion to locate a SNP on Omni2.5 that: (i) is in perfect correlation (*r* ^2^  = 1) with the target SNP in both CEU and JPT+CHB; (ii) is in perfect correlation with the target SNP in only CEU; (iii) is in perfect correlation with the target SNP in only JPT+CHB; (iv) exhibits the highest *r* ^2^ with the target SNP in the combined CEU and JPT+CHB dataset, provided the highest *r* ^2^ is at least 0.80. The search for surrogates considered SNPs on Omni2.5 that were located within 1 Mb of each target SNP. This procedure allowed us to recover at least 470,208 SNPs and 803,333 SNPs on the HumanHap550 and Human1M respectively (see **Table S3**).

### Genotype imputation and haplotype reference maps

The microarray data from the samples were pre-phased using SHAPEIT v2.r644 [20], [21], before imputing with IMPUTE version 2.3.0 [15], [19]. Three reference panels involving the integrated variant set from Phase 1 of the 1KGP with 1,092 individuals were used to perform the imputation: (i) using only the 1KGP reference panel (**Tables S4, S5, S6**); (ii) complementing the 1KGP reference panel with the reference panel from the Singapore Sequencing Malay Project (SSMP) – a population-level deep whole genome sequencing of 96 Southeast Asian Malays in Singapore [22]; and (iii) complementing the 1KGP reference panel with the reference panel from the Singapore Sequencing Indian Project (SSIP) – a population-level deep whole genome sequencing of 36 south Asian Indians in Singapore (in review). The genetic maps from the 1KGP were used for imputation with all three sets of reference panels. The effective population size was set at 15000 to match the parameters used in SHAPEIT, and imputation was performed on chromosomal blocks of 5 Mb while additionally allowing for a buffer size of 5 Mb on each side. The reference panels were merged using the option “merge_ref_panels”, and discrete genotype calls were made by thresholding the imputed genotype posterior probabilities at 0.90. In performing the imputation, only the iOmics samples that were not part of the SSMP and SSIP were used as target populations to be imputed to avoid over-fitting (110 Chinese, 50 Malays and 70 Indians).

### Quantifying imputation quality

To assess whether statistical imputation can successfully recover the unobserved exonic SNPs effectively, we measured the quality of the imputed data at the SNP level and at the individual sample genotype level.

At the SNP level, we considered the information criterion generated by IMPUTE (info) as part of the imputation process. This metric is meant as a measure of the multi-SNP correlation between neighboring SNPs and the target SNP. We defined a SNP to be well-imputed when info was at least 0.3. In addition, we required the imputation to produce high-confidence genotypes for the samples at each SNP. We measured this by calculating the proportion of the samples that carried a valid genotype call, defined as an imputed genotype possessing a posterior probability that was at least 0.90. When all three genotype classes possessed posterior probabilities less than 0.90, a missing genotype was assigned. An imputed SNP was considered to produce high-confidence genotypes when at least 95% of the samples were assigned valid genotypes. Thus a SNP was deemed to be poorly imputed if either info <0.3 or call rate <0.95.

As the info score may not necessarily be an accurate measurement of imputation performance for SNPs with low minor allele frequency, and to capture the situations where the imputation algorithm incorrectly produced high-confidence or high-information output due to the use of an inappropriate reference panel for the target population, we also considered the accuracy of the imputed genotypes by assessing the concordance between the imputed genotypes and the observed genotypes at SNPs that are present on both the reference panel and the exome chip. Each of these SNPs was categorized according to the minor allele frequency (MAF) as rare (MAF ≤0.01), low-frequency (0.01< MAF <0.05) or common (MAF ≥0.05). Two measures of concordance were used: (i) overall concordance, defined as the proportion of imputed genotypes that were identical to the observed genotypes; and (ii) minor allele concordance, defined as the proportion of imputed genotypes with at least 1 minor allele that were identical to the observed genotypes, calculated only across the high-confidence low-frequency and rare SNPS (see **Tables S7, S8, S9** for Omni2.5, **Tables S10, S11, S12** for HumanHap550, **Tables S13, S14, S15** for Human1M on the number of high-confidence imputed SNPs). The motivation behind measuring minor allele concordance is due to the greater emphasis of getting such genotype calls to be accurate for the intended purpose of the exome chip – to locate low-frequency or rare coding SNPs that are associated with phenotypic outcomes.

## Results

Quality checks of the genotype data for the 360 samples in iOmics yielded a final data freeze of 110 Chinese, 108 Malays and 105 Indians with high fidelity data for both Omni2.5 and the exome chip. The number of SNPs after QC ranged between 2,358,215 and 2,358,634 for Omni2.5, and between 272,680 and 272,857 for the exome array, of which there were at least 39,631 SNPs that were present on the exome array and the Omni2.5 (**Table S2**). Of the 323 samples that remained after QC, 93 samples (58 Malays, 35 Indians) were part of the population-level whole genome sequencing of Singapore Malays (SSMP) and Indians (SSIP) and these were excluded from the imputation analyses as they constituted part of the haplotype reference panels used for imputation.

### Polymorphic extent of the exome chip

**Figure 1:**
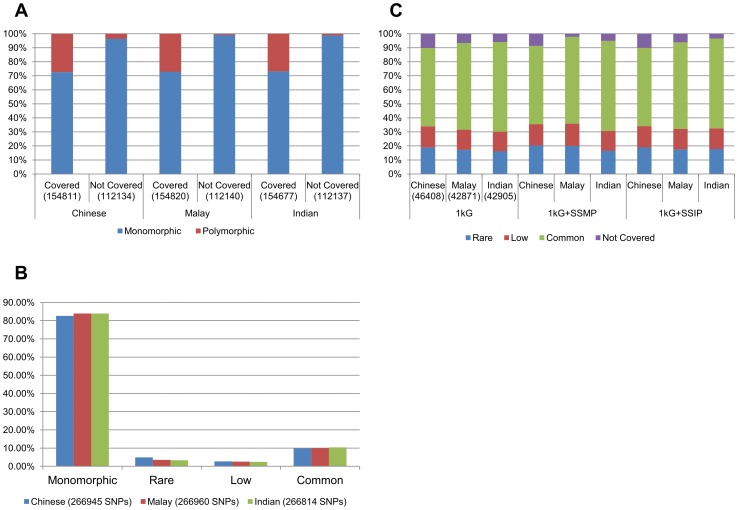
(A) The proportion of monomorphic and polymorphic exonic variants in the Illumina exome chip when assessed in each of the three Singapore populations. The exonic variants on the exome chip are further categorized according to whether they are present in any of the reference panels from the 1000 Genomes Project or the Singapore Sequencing Study for the Malays and Indians (“Covered”) and can in theory be imputed, or not present in any of the existing reference panels and thus cannot be recovered through imputation (“Not covered”). (B) Distribution of SNPs on the exome chip according to the minor allele frequencies (MAFs) into monomorphic (MAF  = 0%), rare (0%< MAF ≤1%), low-frequency (1%< MAF ≤5%) and common (MAF >5%) in each of the three populations. (C) MAF categorization of the polymorphic exome chip SNPs in each of the three populations according to whether these SNPs are present (non-purple bars) or not (purple bars) in the respective reference panels. Numbers in brackets indicate the number of SNPs in the respective categories.

For the SNPs that are present on the exome chip, more than 40% are unique to the exome chip design and are not present on any of the existing microarrays or in the reference panels of the 1KGP (Phase 1), SSMP or SSIP, implying that these are the variants can never be recovered from imputation. However, on the basis of 110 Chinese, 108 Malays and 105 Indians that were genotyped on the exome array, almost all of these variants (>95%) are monomorphic in the three Singapore populations (**Figure 1A**). In fact, we observed that more than 80% of the SNPs on the exome chip were actually monomorphic in the three populations (**Figure 1B**). Of the remaining SNPs that were polymorphic, more than 50% had MAF >0.05 which raised the possibility that they may be recoverable from statistical imputation. In particular, if the focus was only on the polymorphic variants on the exome chip, more than 90% of these variants were present in the 1KGP reference panel (**Figure 1C**). The availability of population-specific reference panels for the Malays and Indians provided additional coverage of polymorphic exome chip variants that were not present in the 1KGP.

### Recovering the exome chip with imputation

Although the majority of the polymorphic variants on the exome chip can be found in haplotype reference panels for imputation, whether these SNPs can be successfully recovered for downstream association analyses depends on the quality and accuracy of the genotype imputation. We investigated the imputation performance for 110 Chinese, 50 Malays and 70 Indians, excluding the 58 Malays and 35 Indians that had been whole-genome sequenced and were used to construct haplotype reference panels for imputation. Each sample was imputed against three reference panels: (i) 1KGP; (ii) 1KGP and SSMP; (iii) 1KGP and SSIP. The SSMP and SSIP consisted of 96 Singapore Malays and 36 Singapore Indians respectively that had been whole-genome sequenced to a target coverage of 30X. Two definitions of imputation success were used.

**Figure 2:**
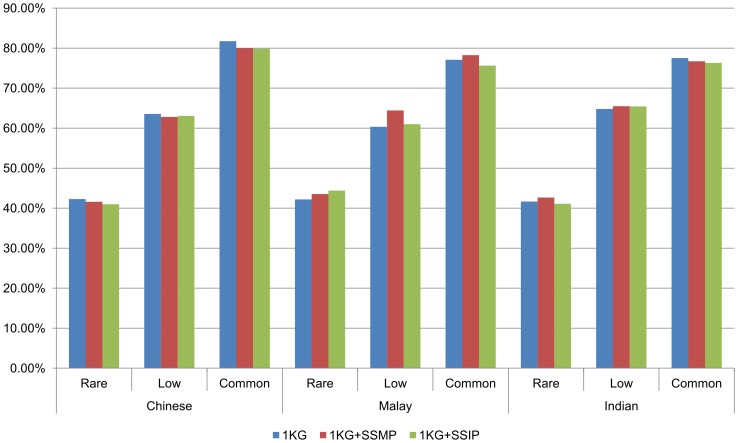
The percentage of polymorphic exome chip SNPs in each of the three populations that can be reliably imputed against three different reference panels using the SNPs on the Illumina HumanOmni2.5 as input. Each of these SNPs is categorized according to the minor allele frequency (MAF) as rare (0%< MAF ≤1%), low-frequency (1%< MAF ≤5%) and common (MAF >5%). See Figures S1 and S2 in the Supplementary Material for the equivalent figures when SNPs on the HumanHap550 and Human1M are used as input respectively. The total number of imputed exome SNPs when using Illumina HumanOmni2.5/HumanHap550/Human1M as the study panel is shown in Table S4, S5 and S6.

A SNP was defined to be successfully imputed if the information metric generated as part of the imputation process was at least 0.3 (IMPUTE info ≥0.30), and if at least 95% of the samples presented a valid genotype call when the imputed genotype posterior probabilities were compared against a threshold of 0.90. The latter metric was akin to a SNP call rate of at least 95%. Jointly these two metrics indicated the extent of correlation between a target SNP with neighboring SNPs, and whether the imputation produced high-confidence calls. As expected, when imputed using the SNPs present on the Omni2.5, in excess of 75% of the common polymorphic SNPs on the exome chip were imputed successfully whereas less than 45% of the rare polymorphic SNPs were successfully imputed regardless of the choice of reference panels (**Figure 2**). Supplementing the 1KGP panel with reference haplotypes from SSMP yielded more successfully imputed SNPs compared to using only the 1KGP panel, although this was not true when the Indians were imputed with a SSIP-supplemented reference panel. Interestingly, even when we downsampled to using only the contents of the HumanHap550 and Human1M, the imputation yielded similar performances in terms of the extent of the rare and low-frequency variants recovered. However, the percentage of common variants recovered decreased by approximately 15% and 10% with the HumanHap550 and Human1M respectively (**Figures S1–S2**).

While it appeared surprising that supplementing 1KGP with the Indian-specific panel did not produce more high quality imputation for the Indians, it was important to note the above two definitions of imputation quality fundamentally relied on the correlation structure between the target SNP and surrounding markers in the reference panel. The imputation algorithm can mistakenly bestow high-confidence calls when the underlying patterns of genetic correlation in the target sample differ considerably from the reference panel. The second measure of imputation success thus focused on the accuracy of the imputed genotypes, evaluated by the degree of concordance between the imputed genotypes and the observed genotypes at SNPs present on the Omni2.5 but absent on the exome chip.

While increasing the size of the 1KGP reference panel with at least 36 more samples improved imputation accuracy, the greatest improvements were seen when the respective population-specific panels were used to supplement the 1KGP to impute the Malays and Indians, where up to 0.55% decrease in discordance was observed for low-frequency SNPs in the Malays when using the Omni2.5 SNPs (**Table 1**). For HumanHap550 and Human1M, the discordance decreased by up to 0.35% and 0.38% for the HumanHap550 and Human1M respectively, **Tables S16–S17**). As the primary intention for the use of the exome chip was to discover phenotype associations with rare and low-frequency SNPs, we additionally evaluated the minor allele discordance – defined as the discordance between imputed and observed genotypes that carried at least one minor allele at rare and low-frequency SNPs. This revealed that supplementing the 1KGP panel with population-specific haplotypes can significantly reduce the error rates of imputing the rare and low-frequency variants by up to 14.86% and 8.44% for the Malays and Indians respectively on the Omni2.5 (**Table 2**), and up to 16.21% and 9.48% respectively even with the lowest density HumanHap550 (**Tables S18**). **Table S19** shows the reduction in error rate with respect to Human1M as the study panel.

**Table 1:** Discordance (%) between imputed genotypes and actually observed genotypes at SNPs on Omni2.5 but not in the exome chip.

Population	SNP Category	Haplotype reference panel for imputation		
		1KGP^1^	1KGP + SSMP^2^	1KGP + SSIP^3^
**Chinese**	Rare	0.78	0.70	0.72
	Low-freq	1.12	1.07	1.07
	Common	0.42	0.39	0.39
**Malay**	Rare	0.75	**0.44**	0.67
	Low-freq	1.40	**0.85**	1.16
	Common	0.80	**0.51**	0.72
**Indian**	Rare	0.77	0.60	**0.59**
	Low-freq	1.16	1.00	**0.89**
	Common	0.86	0.70	**0.60**

1 Phase 1 of the 1KGP, consisting of 1,092 subjects.

2 Singapore Sequencing Malay Project, consisting of 96 Southeast Asian Malays that have been whole-genome sequenced at 30X.

3 Singapore Sequencing Indian Project, consisting of 36 South Asian Indians that have been whole-genome sequenced at 30X.

**Table 2:** Discordance (%) between imputed genotypes and actually observed minor allele genotypes^1^ at rare and low-frequency SNPs on the exome chip but not in the Omni2.5.

Population	SNP Category	Haplotype reference panel for imputation		
		1KGP^2^	1KGP + SSMP^3^	1KGP + SSIP^4^
**Chinese**	Rare	42.93	40.24	41.03
	Low-freq	21.65	21.15	21.33
**Malay**	Rare	27.23	**12.37**	24.61
	Low-freq	20.42	**12.13**	17.82
**Indian**	Rare	28.64	24.31	**20.20**
	Low-freq	18.41	15.53	**14.58**

1 A minor allele genotype is defined as a genotype that carries at least one copy of the minor allele, and discordance here is measured against the total number of observed minor allele genotypes at rare and low-frequency SNPs.

2 Phase 1 of the 1KGP, consisting of 1,092 subjects.

3 Singapore Sequencing Malay Project, consisting of 96 Southeast Asian Malays that have been whole-genome sequenced at 30X.

4 Singapore Sequencing Indian Project, consisting of 36 South Asian Indians that have been whole-genome sequenced at 30X.

### Actual coverage of exome chip

The discussion thus far has focused on quantifying how much of the exome chip was actually polymorphic and the extent that these polymorphic SNPs can be recovered by statistical imputation. It is however just as important to evaluate whether the exome chip is representative of the full set of polymorphic exonic variants in other populations, or whether it is only relevant to the populations that were used to design the chip. For example, the SSMP identified 261,962 SNPs in the exonic regions of which only 28,049 SNPs were present on the exome chip. Similarly, out of 183,835 polymorphic exonic SNPs in SSIP, only 22,039 SNPs were present on the exome chip (**Table S20**). In contrast, 43% of the full set of exonic variants in SSMP (58.42% for SSIP) can be recovered successfully (info ≥0.3 and call rate ≥0.95, including the exonic variants from study sample) by imputing off the 1KGP reference panel (**Table 3**). Even with the lowest density content from the HumanHap550, we can recover 30.93% of the full set of exonic variants in SSMP (42.24% for SSIP, **Table S21**). When a population-specific reference panel of at least 36 individuals was supplemented, the proportion that was successfully recovered increased to 47.20% for SSMP and 62.21% for SSIP (**Table 3**).

**Table 3:** Actual and recoverable content of exonic variants in 96 Malays (SSMP) and 36 Indians (SSIP) based on HumanOmni2.5 as the study panel.

Number of exonic SNPs	SSMP		SSIP	
	Rare/Low-freq	Common	Rare/Low-freq	Common
**In total**	167,523	94,439	91,157	92,678
**Overlap Omni2.5** 1	19,253	47,148	13,839	50,136
**On exome chip**	11,831	13,226	7,920	13,694
**Imputed off 1KGP**	14,622	31,612	12,534	30,880
**Imputed off 1KGP+SSMP**	**24,394**	**32,860**	13,028	30,783
**Imputed off 1KGP+SSIP**	14,961	30,528	**18,345**	**32,042**

1 Overlap Omni2.5 is the total number of exonic variants from SSMP or SSIP that overlaps with genotypes from HumanOmni2.5 array. The number of imputed variants does not include these overlapped variants. For the corresponding results for HumanHap550/Human1M as the study panel, please refer to Table S21 and S22 accordingly.

## Discussion

We have investigated the relevance of the Illumina HumanExome array for three populations in Southeast Asia comprising the Southern Han Chinese, Southeast Asian Malays and South Asian Indians. We observed that more than 80% of the content of the exome chip was monomorphic when assessed in about 100 individuals from each of these populations. Of the remaining SNPs that were polymorphic, at least 55% could be imputed successfully even with the use of early generation GWAS arrays such as the Illumina HumanHap550K or the Illumina Human1M, together with the haplotype reference panel from Phase 1 of the 1KGP. Imputation accuracy was increased by supplementing the 1KGP panel with population-specific whole-genome sequencing of at least 36 individuals. What was striking was the exome chip only provided an actual coverage of 9.57% of the polymorphic exonic variants that were present from whole-genome sequencing a separate collection of 96 Malays (11.76%, for the equivalent in 36 Indians), and imputation against the 1KGP alone could already recover at least 30.93% of these variants successfully with high confidence in the Malay population (42.24% for Indian population) based upon the lowest density content genotyping array HumanHap550. Supplementing the 1KGP reference panel with population-specific content further increased the recoverable coverage to at least 32.44% for Malay population (42.74% for Indian population) using HumanHap550 as study panel, far surpassing the coverage provided by the exome chip (**Table S22**, **Table S19** for the respective information for Human1M).

It is important to note that we have sought to evaluate the representation and coverage of the exome chip in two populations (Malays, Indians) which were not part of the design and for which we have deep whole-genome sequencing data for. We did not investigate whether the conclusions were similarly relevant for European populations, which contributed the greatest resource during the design of the exome chip. However, we similarly noticed that in excess of 80% of the SNP content for the exome chip was monomorphic in the Chinese, despite the inclusion of samples of Chinese ancestry in the design. In addition, we will like to emphasize that our categorization of the SNPs into the four minor allele frequency bins of monomorphic, rare, low-frequency and common were made on the basis of our existing samples, and it is entirely possible that as the number of samples genotyped increases, a fraction of the SNPs currently categorized as monomorphic can actually turn out to be polymorphic albeit with low allele counts.

From the perspective of cost, at the price of US$50 per exome chip (ignoring manpower and other infrastructure expenses) and US$3000 to perform 30-fold whole-genome sequencing of one genome, the cost to genotype 2,100 samples on the exome chip is equivalent to sequencing 35 samples. However, we have shown that the information generated from the whole-genome sequencing of at least 35 samples, together with publicly available information from the 1KGP, delivers a more comprehensive coverage of the exonic variants for the new population than additionally genotyping samples with the exome chip. The sequencing information can also be naturally extended to impute other populations as well, thereby benefitting multiple projects. Based on this assessment, we recommend that it is more cost-effective to perform deep whole-genome sequencing of a small subset of the samples in order to generate population-specific reference haplotype maps to complement the 1KGP, than to extend the use of the exome chip to studies with pre-existing GWAS data.

Our assessment of imputation performance focused on different aspects of quality and accuracy. We particularly highlighted the difference between: (i) SNP-level imputation confidence, as measured by the information content and the peakedness of imputed genotype posterior probabilities (which affects the call rate at a SNP); and (ii) inherent accuracy between the imputed genotypes and the actually observed genotypes. While the latter metric is more appropriate in assessing imputation performance, it is often not possible to evaluate this agnostically as this requires the target set of SNPs to have already been genotyped. As such, the information criterion from imputation is often used as a surrogate measure of imputation quality. Jallow and colleagues illustrated that relying on the information criterion can produce erroneous outcomes, since imputation can mistakenly bestow high-confidence calls when the underlying haplotype structure in a target sample differs from that present in the reference panel [23]. Thus, our evaluation utilized both measures of SNP-level imputation confidence and imputation accuracy in order to provide a more meaningful assessment of the imputation strategy for recovering exome chip content.

In addition to measuring overall accuracy between imputed and observed genotypes, we specifically measured the discordance at observed genotypes that carried at least one minor allele at low-frequency and rare SNPs. There are two reasons here: (i) given the low frequency of the minor alleles at such SNPs, miscalling all the genotypes as major allele homozygotes only contributes a small degree of discordance and this can send the incorrect impression that imputation is highly accurate (i.e. >99% accuracy for a rare variant incorrectly imputed as monomorphic); (ii) the use of the exome chip is primarily intended to identify association with low-frequency and rare SNPs, and erroneously calling the presence or absence of a minor allele genotype can affect the power and false positive rate of the association analyses, especially when it is common to use statistical methods that pool allele counts across multiple SNPs in a gene region. Thus, in our evaluation of the use of imputation to recover exonic SNPs, we specifically assessed the ability to accurately determine the minor allele genotypes.

One criticism to imputing the exonic variants is the need to address imputation uncertainty in during downstream analyses, in order to maximize statistical power [24]. Methodologies such as MACH2qtl [25], ProbABEL [26], SNPTEST [27] and PLINK [28] have the option of utilizing the genotype posterior probabilities or dosages in the association analyses, although these approaches are mostly for testing association at a single SNP and have not been extended to evaluate allele burden across multiple SNPs. However, this seems surmountable from an analytical perspective, particularly when the alternative is to perform additional genotyping on a microarray which provides an effective coverage of less than 10%.

When it comes to querying low-frequency or rare variants in the genome, there is likely to be no cost-effective replacement for sequencing. While genotyping with pre-designed microarrays has offered spectacularly success at representing common genomic content, our evaluation here has shown there is no significant advantage in additionally genotyping samples with GWAS information on the exome chip, over what statistical imputation with existing haplotype reference panels can already provide. If any, performing population-level deep whole-genome sequencing for as many subjects as possible for the cost of genotyping a GWAS cohort on the exome chip may yield greater returns.

## Supporting Information

Figure S1 **The percentage of polymorphic exome chip SNPs in each of the three populations that can be reliably imputed against three different reference panels using the SNPs on the Illumina HumanHap550 as input.** Each of these SNPs is categorized according to the minor allele frequency (MAF) as rare (0%< MAF ≤1%), low-frequency (1%< MAF ≤5%) and common (MAF >5%).
(TIF)

Figure S2 **The percentage of polymorphic exome chip SNPs in each of the three populations that can be reliably imputed against three different reference panels using the SNPs on the Illumina Human1M as input.** Each of these SNPs is categorized according to the minor allele frequency (MAF) as rare (0%< MAF ≤1%), low-frequency (1%< MAF ≤5%) and common (MAF >5%).
(TIF)

Table S1 **Details on samples removed during the quality control process.**
(DOCX)

Table S2 **Number of SNPs remaining after the quality control process, assessed on the basis of the post-QC samples.**
(DOCX)

Table S3 **Number of SNPs available after rebuilding the Illumina HumanHap550 and Human1M from the Omni2.5.**
(DOCX)

Table S4 **Total number of imputed SNPs using 1000 Genome (1KG) Reference panel and Illumina HumanOmni2.5 as the study panel.**
(DOCX)

Table S5 **Total number of imputed SNPs using 1000 Genome (1KG) Reference panel and rebuilt Illumina HumanHap550 as the study panel.**
(DOCX)

Table S6 **Total number of imputed SNPs using 1000 Genome (1KG) Reference panel and rebuilt Illumina Human1M as the study panel.**
(DOCX)

Table S7 **Total number of imputed exome SNPs with info ≥0.3 that have call rate ≥95% in the Chinese, based on the SNPs on the Omni2.5.**
(DOCX)

Table S8 **Total number of imputed exome SNPs with info ≥0.3 that have call rate ≥95% in the Malays, based on the SNPs on the Omni2.5.**
(DOCX)

Table S9 **Total number of imputed exome SNPs with info ≥0.3 that have call rate ≥95% in the Indians, based on the SNPs on the Omni2.5.**
(DOCX)

Table S10 **Total number of imputed exome SNPs with info ≥0.3 that have call rate ≥95% in the Chinese, based on the SNPs on the HumanHap550.**
(DOCX)

Table S11 **Total number of imputed exome SNPs with info ≥0.3 that have call rate ≥95% in the Malays, based on the SNPs on the HumanHap550.**
(DOCX)

Table S12 **Total number of imputed exome SNPs with info ≥0.3 that have call rate ≥95% in the Indians, based on the SNPs on the HumanHap550.**
(DOCX)

Table S13 **Total number of imputed exome SNPs with info ≥0.3 that have call rate ≥95% in the Chinese, based on the SNPs on the Human1M.**
(DOCX)

Table S14 **Total number of imputed exome SNPs with info ≥0.3 that have call rate ≥95% in the Malays, based on the SNPs on the Human1M.**
(DOCX)

Table S15 **Total number of imputed exome SNPs with info ≥0.3 that have call rate ≥95% in the Indians, based on the SNPs on the Human1M.**
(DOCX)

Table S16 **Discordance (%) between imputed genotypes and actually observed genotypes at highly reliably imputed exome SNPs using HumanHap550 as the study panel.** ^1^ Phase 1 of the 1KGP, consisting of 1,092 subjects. ^2^ Singapore Sequencing Malay Project, consisting of 96 Southeast Asian Malays that have been whole-genome sequenced at 30X. ^3^ Singapore Sequencing Indian Project, consisting of 36 South Asian Indians that have been whole-genome sequenced at 30X.
(DOCX)

Table S17 **Discordance (%) between imputed genotypes and actually observed genotypes at highly reliably imputed exome SNPs using Human1M as the study panel.** ^1^ Phase 1 of the 1KGP, consisting of 1,092 subjects. ^2^ Singapore Sequencing Malay Project, consisting of 96 Southeast Asian Malays that have been whole-genome sequenced at 30X. ^3^ Singapore Sequencing Indian Project, consisting of 36 South Asian Indians that have been whole-genome sequenced at 30X.
(DOCX)

Table S18 **Discordance (%) between imputed genotypes and actually observed minor allele genotypes^1^ at rare and low-frequency SNPs using HumanHap550 as the study panel.** ^1^ A minor allele genotype is defined as a genotype that carries at least one copy of the minor allele, and discordance here is measured against the total number of observed minor allele genotypes at rare and low-frequency SNPs. ^2^ Phase 1 of the 1KGP, consisting of 1,092 subjects. ^3^ Singapore Sequencing Malay Project, consisting of 96 Southeast Asian Malays that have been whole-genome sequenced at 30X. ^4^ Singapore Sequencing Indian Project, consisting of 36 South Asian Indians that have been whole-genome sequenced at 30X.
(DOCX)

Table S19 **Discordance (%) between imputed genotypes and actually observed minor allele genotypes^1^ at rare and low-frequency SNPs using Human1M as the study panel.** ^1^ A minor allele genotype is defined as a genotype that carries at least one copy of the minor allele, and discordance here is measured against the total number of observed minor allele genotypes at rare and low-frequency SNPs. ^2^ Phase 1 of the 1KGP, consisting of 1,092 subjects. ^3^ Singapore Sequencing Malay Project, consisting of 96 Southeast Asian Malays that have been whole-genome sequenced at 30X. ^4^ Singapore Sequencing Indian Project, consisting of 36 South Asian Indians that have been whole-genome sequenced at 30X.
(DOCX)

Table S20 **Number of overlapping exonic exome variants with whole genome sequencing data.** ^1^All exonic exome: Malay  = 249,940; Indian  = 249,821. ^2^Polymorphic exonic exome: Malay  = 28,528; Indian  = 28,474. ^3^Proportion of polymorphic exonic exome is defined as the number of overlap polymorphic exonic exome divide by the total number of exonic SSMP/SSIP respectively. ^4^SSMP and SSIP variants are all polymorphic.
(DOCX)

Table S21 **Actual and recoverable content of exonic variants in 96 Malays (SSMP) and 36 Indians (SSIP) based on HumanHap550 as the study panel.** ^1^Overlap HumanHap550 is the total number of exonic variants from SSMP or SSIP that overlaps with genotypes from HumanHap550 array. The number of imputed variants does not include these overlapped variants.
(DOCX)

Table S22 **Actual and recoverable content of exonic variants in 96 Malays (SSMP) and 36 Indians (SSIP) based on Human1M as the study panel.** ^1^Overlap Human1M is the total number of exonic variants from SSMP or SSIP that overlaps with genotypes from Human1M array. The number of imputed variants does not include these overlapped variants.
(DOCX)
