# Supplementary figures and images for: Evaluating the Coverage and Potential of Imputing the Exome Microarray with Next-Generation Imputation Using the 1000 Genomes Project
Source: PLoS One. 2014 Sep 9;9(9):e106681. doi: 10.1371/journal.pone.0106681 (PMC4159276; doi:10.1371/journal.pone.0106681)

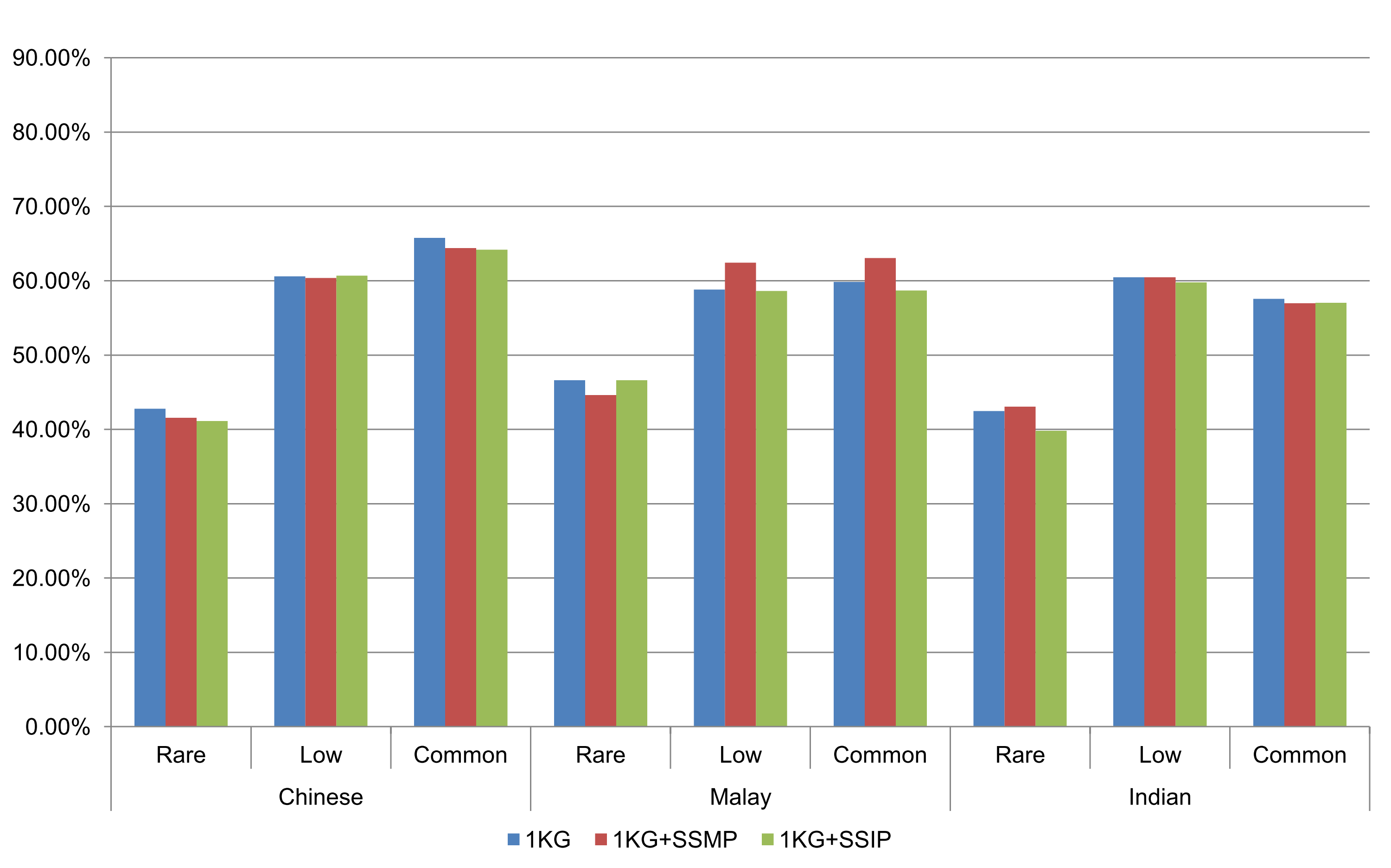

Supplement: Figure S1 — The percentage of polymorphic exome chip SNPs in each of the three populations that can be reliably imputed against three different reference panels using the SNPs on the Illumina HumanHap550 as input. Each of these SNPs is categorized according to the minor allele frequency (MAF) as rare (0%< MAF ≤1%), low-frequency (1%< MAF ≤5%) and common (MAF >5%). (TIF) [file pone.0106681.s001.tif]

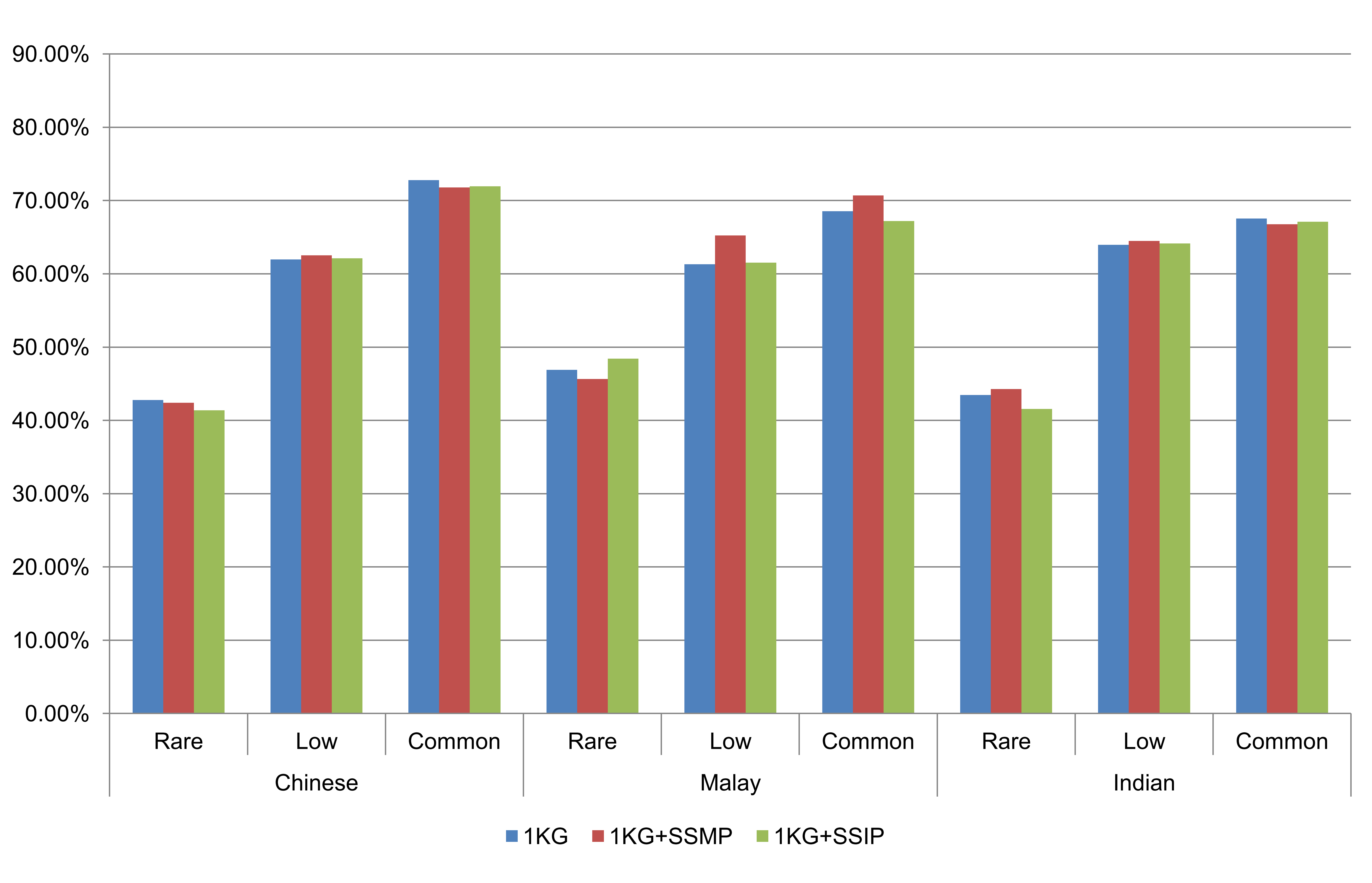

Supplement: Figure S2 — The percentage of polymorphic exome chip SNPs in each of the three populations that can be reliably imputed against three different reference panels using the SNPs on the Illumina Human1M as input. Each of these SNPs is categorized according to the minor allele frequency (MAF) as rare (0%< MAF ≤1%), low-frequency (1%< MAF ≤5%) and common (MAF >5%). (TIF) [file pone.0106681.s002.tif]
