# Supplementary material for: Evaluating the Coverage and Potential of Imputing the Exome Microarray with Next-Generation Imputation Using the 1000 Genomes Project
Source: PLoS One. 2014 Sep 9;9(9):e106681. doi: 10.1371/journal.pone.0106681 (PMC4159276; doi:10.1371/journal.pone.0106681)
Supplement: Table S1 — Details on samples removed during the quality control process. (DOCX) [file pone.0106681.s003.docx]

**Table S1.** Details on samples removed during the quality control process

| **QC criterion** | **Chinese (111)** | **Malays (120)** | **Indians (119)** |
| --- | --- | --- | --- |
| **High Missingness (>2%)** | - | - | 1 |
| **Excessive IBS** | - | 7 | 8 |
| **PCA outliers** | 1 | 5 | 5 |
| **Remaining #samples** | 110 | 108 | 105 |
