# Supplementary material for: Evaluating the Coverage and Potential of Imputing the Exome Microarray with Next-Generation Imputation Using the 1000 Genomes Project
Source: PLoS One. 2014 Sep 9;9(9):e106681. doi: 10.1371/journal.pone.0106681 (PMC4159276; doi:10.1371/journal.pone.0106681)
Supplement: Table S2 — Number of SNPs remaining after the quality control process, assessed on the basis of the post-QC samples. (DOCX) [file pone.0106681.s004.docx]

**Table S2.** Number of SNPs remaining after the quality control process, assessed on the basis of the post-QC samples

| **Population** | **Illumina HumanOmni2.5** | **Illumina HumanExome** | **# SNPs Overlap** |
| --- | --- | --- | --- |
| **110 Chinese** | 2,358,634 | 272,837 | 39,635 |
| **108 Malay** | 2,358,620 | 272,857 | 39,649 |
| **105 Indian** | 2,358,215 | 272,680 | 39,631 |
