# Supplementary material for: Evaluating the Coverage and Potential of Imputing the Exome Microarray with Next-Generation Imputation Using the 1000 Genomes Project
Source: PLoS One. 2014 Sep 9;9(9):e106681. doi: 10.1371/journal.pone.0106681 (PMC4159276; doi:10.1371/journal.pone.0106681)
Supplement: Table S3 — Number of SNPs available after rebuilding the Illumina HumanHap550 and Human1M from the Omni2.5. (DOCX) [file pone.0106681.s005.docx]

**Table S3.** Number of SNPs available after rebuilding the Illumina HumanHap550 and Human1M from the Omni2.5

| **Population** | **HumanHap550** | | **Human1M** | |
| --- | --- | --- | --- | --- |
|  | Overlap | Overlap+Surrogate | Overlap | Overlap+Surrogate |
| **110 Chinese** | 377,563 | 470,256 | 681,328 | 803,441 |
| **108 Malay** | 377,563 | 470,276 | 681,328 | 803,476 |
| **105 Indian** | 377,563 | 470,208 | 681,328 | 803,333 |
