# Supplementary material for: Evaluating the Coverage and Potential of Imputing the Exome Microarray with Next-Generation Imputation Using the 1000 Genomes Project
Source: PLoS One. 2014 Sep 9;9(9):e106681. doi: 10.1371/journal.pone.0106681 (PMC4159276; doi:10.1371/journal.pone.0106681)
Supplement: Table S4 — Total number of imputed SNPs using 1000 Genome (1KG) Reference panel and Illumina HumanOmni2.5 as the study panel. (DOCX) [file pone.0106681.s006.docx]

**Table S4.** Total number of imputed SNPs using 1000 Genome (1KG) Reference panel and Illumina HumanOmni2.5 as the study panel

| **Category** | **Chinese** | **Malay** | **Indian** |
| --- | --- | --- | --- |
| # Rare (0 < x ≤ 1%) | 7,392 | 3,090 | 3,168 |
| # Low (1% < x < 5%) | 4,169 | 2,438 | 2,995 |
| # Common (≥ 5%) | 5,815 | 6,214 | 6,296 |
| **Total** | **17,376** | **11,742** | **12,459** |
