# Supplementary material for: Evaluating the Coverage and Potential of Imputing the Exome Microarray with Next-Generation Imputation Using the 1000 Genomes Project
Source: PLoS One. 2014 Sep 9;9(9):e106681. doi: 10.1371/journal.pone.0106681 (PMC4159276; doi:10.1371/journal.pone.0106681)
Supplement: Table S6 — Total number of imputed SNPs using 1000 Genome (1KG) Reference panel and rebuilt Illumina Human1M as the study panel. (DOCX) [file pone.0106681.s008.docx]

**Table S6.** Total number of imputed SNPs using 1000 Genome (1KG) Reference panel and rebuilt Illumina Human1M as the study panel

| **Category** | **Chinese** | **Malay** | **Indian** |
| --- | --- | --- | --- |
| # Rare (0 < x ≤ 1%) | 8,009 | 3,872 | 3,972 |
| # Low (1% < x < 5%) | 5,287 | 3,275 | 4,369 |
| # Common (≥ 5%) | 10,118 | 10,612 | 10,840 |
| **Total** | **23,414** | **17,759** | **19,181** |
