# Supplementary material for: Evaluating the Coverage and Potential of Imputing the Exome Microarray with Next-Generation Imputation Using the 1000 Genomes Project
Source: PLoS One. 2014 Sep 9;9(9):e106681. doi: 10.1371/journal.pone.0106681 (PMC4159276; doi:10.1371/journal.pone.0106681)
Supplement: Table S7 — Total number of imputed exome SNPs with info ≥0.3 that have call rate ≥95% in the Chinese, based on the SNPs on the Omni2.5. (DOCX) [file pone.0106681.s009.docx]

**Table S7.** Total number of imputed exome SNPs with info ≥ 0.3 that have call rate ≥ 95% in the Chinese, based on the SNPs on the Omni2.5

| **Category** | **1KG** | **1KG+SSMP** | **1KG+SSIP** |
| --- | --- | --- | --- |
| # Rare (0 < x ≤ 1%) | 3,125 | 3,074 | 3,028 |
| # Low (1% < x < 5%) | 2,649 | 2,618 | 2,629 |
| # Common (≥ 5%) | 4,753 | 4,651 | 4,642 |
| **Total** | **10,527** | **10,343** | **10,299** |
