# Supplementary material for: Evaluating the Coverage and Potential of Imputing the Exome Microarray with Next-Generation Imputation Using the 1000 Genomes Project
Source: PLoS One. 2014 Sep 9;9(9):e106681. doi: 10.1371/journal.pone.0106681 (PMC4159276; doi:10.1371/journal.pone.0106681)
Supplement: Table S8 — Total number of imputed exome SNPs with info ≥0.3 that have call rate ≥95% in the Malays, based on the SNPs on the Omni2.5. (DOCX) [file pone.0106681.s010.docx]

**Table S8** Total number of imputed exome SNPs with info ≥ 0.3 that have call rate ≥ 95% in the Malays, based on the SNPs on the Omni2.5

| **Category** | **1KG** | **1KG+SSMP** | **1KG+SSIP** |
| --- | --- | --- | --- |
| # Rare (0 < x ≤ 1%) | 1,304 | 1,345 | 1,372 |
| # Low (1% < x < 5%) | 1,471 | 1,571 | 1,487 |
| # Common (≥ 5%) | 4,790 | 4,862 | 4,700 |
| **Total** | **7,565** | **7,778** | **7,559** |
