# Supplementary material for: Evaluating the Coverage and Potential of Imputing the Exome Microarray with Next-Generation Imputation Using the 1000 Genomes Project
Source: PLoS One. 2014 Sep 9;9(9):e106681. doi: 10.1371/journal.pone.0106681 (PMC4159276; doi:10.1371/journal.pone.0106681)
Supplement: Table S10 — Total number of imputed exome SNPs with info ≥0.3 that have call rate ≥95% in the Chinese, based on the SNPs on the HumanHap550. (DOCX) [file pone.0106681.s012.docx]

**Table S10.** Total number of imputed exome SNPs with info ≥ 0.3 that have call rate ≥ 95% in the Chinese, based on the SNPs on the HumanHap550

| **Category** | **1KG** | **1KG+SSMP** | **1KG+SSIP** |
| --- | --- | --- | --- |
| # Rare (0 < x ≤ 1%) | 3,627 | 3,524 | 3,488 |
| # Low (1% < x < 5%) | 3,757 | 3,745 | 3,763 |
| # Common (≥ 5%) | 10,260 | 10,047 | 10,013 |
| **Total** | **17,644** | **17,316** | **17,264** |
| **Overlap Omni2.5** | **8,470** | **8,382** | **8,343** |
| **After excluding Omni2.5 SNPs** | **9,174** | **8,934** | **8,921** |
