# Supplementary material for: Evaluating the Coverage and Potential of Imputing the Exome Microarray with Next-Generation Imputation Using the 1000 Genomes Project
Source: PLoS One. 2014 Sep 9;9(9):e106681. doi: 10.1371/journal.pone.0106681 (PMC4159276; doi:10.1371/journal.pone.0106681)
Supplement: Table S11 — Total number of imputed exome SNPs with info ≥0.3 that have call rate ≥95% in the Malays, based on the SNPs on the HumanHap550. (DOCX) [file pone.0106681.s013.docx]

**Table S11.** Total number of imputed exome SNPs with info ≥ 0.3 that have call rate ≥ 95% in the Malays, based on the SNPs on the HumanHap550

| **Category** | **1KG** | **1KG+SSMP** | **1KG+SSIP** |
| --- | --- | --- | --- |
| # Rare (0 < x ≤ 1%) | 2,039 | 1,951 | 2,038 |
| # Low (1% < x < 5%) | 2,357 | 2,502 | 2,350 |
| # Common (≥ 5%) | 9,713 | 10,233 | 9,525 |
| **Total** | **14,109** | **14,686** | **13,913** |
| **Overlap Omni2.5** | **7,842** | **8,242** | **7,709** |
| **After excluding Omni2.5 SNPs** | **6,267** | **6,444** | **6,204** |
