# Supplementary material for: Evaluating the Coverage and Potential of Imputing the Exome Microarray with Next-Generation Imputation Using the 1000 Genomes Project
Source: PLoS One. 2014 Sep 9;9(9):e106681. doi: 10.1371/journal.pone.0106681 (PMC4159276; doi:10.1371/journal.pone.0106681)
Supplement: Table S12 — Total number of imputed exome SNPs with info ≥0.3 that have call rate ≥95% in the Indians, based on the SNPs on the HumanHap550. (DOCX) [file pone.0106681.s014.docx]

**Table S12.** Total number of imputed exome SNPs with info ≥ 0.3 that have call rate ≥ 95% in the Indians, based on the SNPs on the HumanHap550

| **Category** | **1KG** | **1KG+SSMP** | **1KG+SSIP** |
| --- | --- | --- | --- |
| # Rare (0 < x ≤ 1%) | 1,885 | 1,912 | 1,767 |
| # Low (1% < x < 5%) | 3,264 | 3,263 | 3,226 |
| # Common (≥ 5%) | 9,567 | 9,471 | 9,483 |
| **Total** | **14,716** | **14,646** | **14,476** |
| **Overlap Omni2.5** | **8,175** | **8,098** | **8,081** |
| **After excluding Omni2.5 SNPs** | **6,541** | **6,548** | **6,395** |
