# Supplementary material for: Evaluating the Coverage and Potential of Imputing the Exome Microarray with Next-Generation Imputation Using the 1000 Genomes Project
Source: PLoS One. 2014 Sep 9;9(9):e106681. doi: 10.1371/journal.pone.0106681 (PMC4159276; doi:10.1371/journal.pone.0106681)
Supplement: Table S13 — Total number of imputed exome SNPs with info ≥0.3 that have call rate ≥95% in the Chinese, based on the SNPs on the Human1M. (DOCX) [file pone.0106681.s015.docx]

**Table S13.** Total number of imputed exome SNPs with info ≥ 0.3 that have call rate ≥ 95% in the Chinese, based on the SNPs on the Human1M

| **Category** | **1KG** | **1KG+SSMP** | **1KG+SSIP** |
| --- | --- | --- | --- |
| # Rare (0 < x ≤ 1%) | 3,425 | 3,396 | 3,313 |
| # Low (1% < x < 5%) | 3,276 | 3,306 | 3,285 |
| # Common (≥ 5%) | 7,365 | 7,264 | 7,279 |
| **Total** | **14,066** | **13,966** | **13,877** |
| **Overlap Omni2.5** | **4,132** | **4,106** | **4,098** |
| **After excluding Omni2.5 SNPs** | **9,934** | **9,860** | **9,779** |
