# Supplementary material for: Evaluating the Coverage and Potential of Imputing the Exome Microarray with Next-Generation Imputation Using the 1000 Genomes Project
Source: PLoS One. 2014 Sep 9;9(9):e106681. doi: 10.1371/journal.pone.0106681 (PMC4159276; doi:10.1371/journal.pone.0106681)
Supplement: Table S14 — Total number of imputed exome SNPs with info ≥0.3 that have call rate ≥95% in the Malays, based on the SNPs on the Human1M. (DOCX) [file pone.0106681.s016.docx]

**Table S14.** Total number of imputed exome SNPs with info ≥ 0.3 that have call rate ≥ 95% in the Malays, based on the SNPs on the Human1M

| **Category** | **1KG** | **1KG+SSMP** | **1KG+SSIP** |
| --- | --- | --- | --- |
| # Rare (0 < x ≤ 1%) | 1,816 | 1,768 | 1,875 |
| # Low (1% < x < 5%) | 2,008 | 2,137 | 2,015 |
| # Common (≥ 5%) | 7,273 | 7,504 | 7,130 |
| **Total** | **11,097** | **11,409** | **11,020** |
| **Overlap Omni2.5** | **3,953** | **4,130** | **3,951** |
| **After excluding Omni2.5 SNPs** | **7,144** | **7,279** | **7,069** |
