# Supplementary material for: Evaluating the Coverage and Potential of Imputing the Exome Microarray with Next-Generation Imputation Using the 1000 Genomes Project
Source: PLoS One. 2014 Sep 9;9(9):e106681. doi: 10.1371/journal.pone.0106681 (PMC4159276; doi:10.1371/journal.pone.0106681)
Supplement: Table S15 — Total number of imputed exome SNPs with info ≥0.3 that have call rate ≥95% in the Indians, based on the SNPs on the Human1M. (DOCX) [file pone.0106681.s017.docx]

**Table S15.** Total number of imputed exome SNPs with info ≥ 0.3 that have call rate ≥ 95% in the Indians, based on the SNPs on the Human1M

| **Category** | **1KG** | **1KG+SSMP** | **1KG+SSIP** |
| --- | --- | --- | --- |
| # Rare (0 < x ≤ 1%) | 1,727 | 1,759 | 1,651 |
| # Low (1% < x < 5%) | 2,794 | 2,818 | 2,802 |
| # Common (≥ 5%) | 7,323 | 7,238 | 7,274 |
| **Total** | **11,844** | **11,815** | **11,727** |
| **Overlap Omni2.5** | **4,283** | **4,266** | **4,260** |
| **After excluding Omni2.5 SNPs** | **7,561** | **7,549** | **7,467** |
