# Supplementary material for: Evaluating the Coverage and Potential of Imputing the Exome Microarray with Next-Generation Imputation Using the 1000 Genomes Project
Source: PLoS One. 2014 Sep 9;9(9):e106681. doi: 10.1371/journal.pone.0106681 (PMC4159276; doi:10.1371/journal.pone.0106681)
Supplement: Table S16 — Discordance (%) between imputed genotypes and actually observed genotypes at highly reliably imputed exome SNPs using HumanHap550 as the study panel. 1 Phase 1 of the 1KGP, consisting of 1,092 subjects. 2 Singapore Sequencing Malay Project, consisting of 96 Southeast Asian Malays that have been whole-genome sequenced at 30X. 3 Singapore Sequencing Indian Project, consisting of 36 South Asian Indians that have been whole-genome sequenced at 30X. (DOCX) [file pone.0106681.s018.docx]

**Table S16.** Discordance (%) between imputed genotypes and actually observed genotypes at highly reliably imputed exome SNPs using HumanHap550 as the study panel

| **Population** | **SNP Category** | **Haplotype reference panel for imputation** | | |
| --- | --- | --- | --- | --- |
|  |  | **1KGP^1^** | **1KGP + SSMP^2^** | **1KGP + SSIP^3^** |
| **Chinese** | Rare | 0.79 | 0.71 | 0.73 |
|  | Low-freq | 1.10 | 1.03 | 1.08 |
|  | Common | 0.50 | 0.44 | 0.45 |
| **Malay** | Rare | 0.83 | **0.48** | 0.70 |
|  | Low-freq | 1.18 | **0.94** | 1.16 |
|  | Common | 0.95 | **0.62** | 0.82 |
| **Indian** | Rare | 0.82 | 0.63 | **0.64** |
|  | Low-freq | 1.23 | 1.06 | **0.96** |
|  | Common | 1.01 | 0.81 | **0.71** |

^1^ Phase 1 of the 1KGP, consisting of 1,092 subjects.

^2^ Singapore Sequencing Malay Project, consisting of 96 Southeast Asian Malays that have been whole-genome sequenced at 30X.

^3^ Singapore Sequencing Indian Project, consisting of 36 South Asian Indians that have been whole-genome sequenced at 30X.
