# Supplementary material for: Evaluating the Coverage and Potential of Imputing the Exome Microarray with Next-Generation Imputation Using the 1000 Genomes Project
Source: PLoS One. 2014 Sep 9;9(9):e106681. doi: 10.1371/journal.pone.0106681 (PMC4159276; doi:10.1371/journal.pone.0106681)
Supplement: Table S17 — Discordance (%) between imputed genotypes and actually observed genotypes at highly reliably imputed exome SNPs using Human1M as the study panel. 1 Phase 1 of the 1KGP, consisting of 1,092 subjects. 2 Singapore Sequencing Malay Project, consisting of 96 Southeast Asian Malays that have been whole-genome sequenced at 30X. 3 Singapore Sequencing Indian Project, consisting of 36 South Asian Indians that have been whole-genome sequenced at 30X. (DOCX) [file pone.0106681.s019.docx]

**Table S17. Discordance** (%) between imputed genotypes and actually observed genotypes at highly reliably imputed exome SNPs using Human1M as the study panel

| **Population** | **SNP Category** | **Haplotype reference panel for imputation** | | |
| --- | --- | --- | --- | --- |
|  |  | **1KGP^1^** | **1KGP + SSMP^2^** | **1KGP + SSIP^3^** |
| **Chinese** | Rare | 0.80 | 0.71 | 0.74 |
|  | Low-freq | 1.11 | 1.06 | 1.10 |
|  | Common | 0.43 | 0.39 | 0.40 |
| **Malay** | Rare | 0.81 | **0.44** | 0.68 |
|  | Low-freq | 1.22 | **0.84** | 1.10 |
|  | Common | 0.84 | **0.57** | 0.74 |
| **Indian** | Rare | 0.81 | 0.61 | **0.58** |
|  | Low-freq | 1.16 | 0.98 | **0.89** |
|  | Common | 0.91 | 0.74 | **0.64** |

^1^ Phase 1 of the 1KGP, consisting of 1,092 subjects.

^2^ Singapore Sequencing Malay Project, consisting of 96 Southeast Asian Malays that have been whole-genome sequenced at 30X.

^3^ Singapore Sequencing Indian Project, consisting of 36 South Asian Indians that have been whole-genome sequenced at 30X.
