# Supplementary material for: Evaluating the Coverage and Potential of Imputing the Exome Microarray with Next-Generation Imputation Using the 1000 Genomes Project
Source: PLoS One. 2014 Sep 9;9(9):e106681. doi: 10.1371/journal.pone.0106681 (PMC4159276; doi:10.1371/journal.pone.0106681)
Supplement: Table S18 — Discordance (%) between imputed genotypes and actually observed minor allele genotypes1 at rare and low-frequency SNPs using HumanHap550 as the study panel. 1 A minor allele genotype is defined as a genotype that carries at least one copy of the minor allele, and discordance here is measured against the total number of observed minor allele genotypes at rare and low-frequency SNPs. 2 Phase 1 of the 1KGP, consisting of 1,092 subjects. 3 Singapore Sequencing Malay Project, consisting of 96 Southeast Asian Malays that have been whole-genome sequenced at 30X. 4 Singapore Sequencing Indian Project, consisting of 36 South Asian Indians that have been whole-genome sequenced at 30X. (DOCX) [file pone.0106681.s020.docx]

**Table S18.** Discordance (%) between imputed genotypes and actually observed minor allele genotypes^1^ at rare and low-frequency SNPs using HumanHap550 as the study panel

| **Population** | **SNP Category** | **Haplotype reference panel for imputation** | | |
| --- | --- | --- | --- | --- |
|  |  | **1KGP^2^** | **1KGP + SSMP^3^** | **1KGP + SSIP^4^** |
| **Chinese** | Rare | 50.05 | 47.62 | 48.10 |
|  | Low-freq | 20.05 | 20.12 | 21.14 |
| **Malay** | Rare | 31.85 | **15.64** | 28.68 |
|  | Low-freq | 20.01 | **14.26** | 18.82 |
| **Indian** | Rare | 35.39 | 32.13 | **25.91** |
|  | Low-freq | 20.34 | 17.78 | **15.94** |

^1^ A minor allele genotype is defined as a genotype that carries at least one copy of the minor allele, and discordance here is measured against the total number of observed minor allele genotypes at rare and low-frequency SNPs.

^2^ Phase 1 of the 1KGP, consisting of 1,092 subjects.

^3^ Singapore Sequencing Malay Project, consisting of 96 Southeast Asian Malays that have been whole-genome sequenced at 30X.

^4^ Singapore Sequencing Indian Project, consisting of 36 South Asian Indians that have been whole-genome sequenced at 30X.
