# Supplementary material for: Evaluating the Coverage and Potential of Imputing the Exome Microarray with Next-Generation Imputation Using the 1000 Genomes Project
Source: PLoS One. 2014 Sep 9;9(9):e106681. doi: 10.1371/journal.pone.0106681 (PMC4159276; doi:10.1371/journal.pone.0106681)
Supplement: Table S19 — Discordance (%) between imputed genotypes and actually observed minor allele genotypes1 at rare and low-frequency SNPs using Human1M as the study panel. 1 A minor allele genotype is defined as a genotype that carries at least one copy of the minor allele, and discordance here is measured against the total number of observed minor allele genotypes at rare and low-frequency SNPs. 2 Phase 1 of the 1KGP, consisting of 1,092 subjects. 3 Singapore Sequencing Malay Project, consisting of 96 Southeast Asian Malays that have been whole-genome sequenced at 30X. 4 Singapore Sequencing Indian Project, consisting of 36 South Asian Indians that have been whole-genome sequenced at 30X. (DOCX) [file pone.0106681.s021.docx]

**Table S19.** Discordance (%) between imputed genotypes and actually observed minor allele genotypes^1^ at rare and low-frequency SNPs using Human1M as the study panel

| **Population** | **SNP Category** | **Haplotype reference panel for imputation** | | |
| --- | --- | --- | --- | --- |
|  |  | **1KGP^2^** | **1KGP + SSMP^3^** | **1KGP + SSIP^4^** |
| **Chinese** | Rare | 46.74 | 44.71 | 45.16 |
|  | Low-freq | 20.44 | 20.23 | 21.13 |
| **Malay** | Rare | 27.96 | **13.30** | 27.85 |
|  | Low-freq | 18.49 | **11.88** | 15.37 |
| **Indian** | Rare | 31.53 | 27.85 | **21.50** |
|  | Low-freq | 18.51 | 15.37 | **14.55** |

^1^ A minor allele genotype is defined as a genotype that carries at least one copy of the minor allele, and discordance here is measured against the total number of observed minor allele genotypes at rare and low-frequency SNPs.

^2^ Phase 1 of the 1KGP, consisting of 1,092 subjects.

^3^ Singapore Sequencing Malay Project, consisting of 96 Southeast Asian Malays that have been whole-genome sequenced at 30X.

^4^ Singapore Sequencing Indian Project, consisting of 36 South Asian Indians that have been whole-genome sequenced at 30X.
