# Supplementary material for: Evaluating the Coverage and Potential of Imputing the Exome Microarray with Next-Generation Imputation Using the 1000 Genomes Project
Source: PLoS One. 2014 Sep 9;9(9):e106681. doi: 10.1371/journal.pone.0106681 (PMC4159276; doi:10.1371/journal.pone.0106681)
Supplement: Table S20 — Number of overlapping exonic exome variants with whole genome sequencing data. 1All exonic exome: Malay = 249,940; Indian = 249,821. 2Polymorphic exonic exome: Malay = 28,528; Indian = 28,474. 3Proportion of polymorphic exonic exome is defined as the number of overlap polymorphic exonic exome divide by the total number of exonic SSMP/SSIP respectively. 4SSMP and SSIP variants are all polymorphic. (DOCX) [file pone.0106681.s022.docx]

**Table S20.** Number of overlapping exonic exome variants with whole genome sequencing data.

| **Remarks** | **Exonic SSMP^4^ (N=261,962)** | **Exonic SSIP^4^ (N=183,835)** |
| --- | --- | --- |
| Overlap All Exonic Exome^1^ | 28,049 | 22,039 |
| Overlap Polymorphic Exonic Exome^2^ | 25,057 | 21,614 |
| Proportion of Polymorphic Exonic Exome^3^ | 9.57% | 11.76% |

^1^All exonic exome: Malay = 249,940; Indian = 249,821

^2^Polymorphic exonic exome: Malay = 28,528; Indian = 28,474

^3^Proportion of polymorphic exonic exome is defined as the number of overlap polymorphic exonic exome divide by the total number of exonic SSMP/SSIP respectively.

^4^SSMP and SSIP variants are all polymorphic
