# Supplementary material for: Evaluating the Coverage and Potential of Imputing the Exome Microarray with Next-Generation Imputation Using the 1000 Genomes Project
Source: PLoS One. 2014 Sep 9;9(9):e106681. doi: 10.1371/journal.pone.0106681 (PMC4159276; doi:10.1371/journal.pone.0106681)
Supplement: Table S22 — Actual and recoverable content of exonic variants in 96 Malays (SSMP) and 36 Indians (SSIP) based on Human1M as the study panel. 1Overlap Human1M is the total number of exonic variants from SSMP or SSIP that overlaps with genotypes from Human1M array. The number of imputed variants does not include these overlapped variants. (DOCX) [file pone.0106681.s024.docx]

**Table S22.** Actual and recoverable content of exonic variants in 96 Malays (SSMP) and 36 Indians (SSIP) based on Human1M as the study panel

| **Number of exonic SNPs** | **SSMP (261,962)** | | **SSIP (183,835)** | |
| --- | --- | --- | --- | --- |
|  | **Rare/Low-freq** | **Common** | **Rare/Low-freq** | **Common** |
| **In total** | 167,523 | 94,439 | 91,157 | 92,678 |
| **Overlap Human1M**^1^ | 8,926 | 35,504 | 6,097 | 37,542 |
| **On exome chip** | 11,831 | 13,226 | 7,920 | 13,694 |
| **Imputed off 1KGP** | 17,694 | 37,070 | 15,785 | 36,662 |
| **Imputed off 1KGP+SSMP** | **19,779** | **38,395** | 15,736 | 36,310 |
| **Imputed off 1KGP+SSIP** | 17,749 | 35,905 | **16,338** | **37,340** |

^1^Overlap Human1M is the total number of exonic variants from SSMP or SSIP that overlaps with genotypes from Human1M array. The number of imputed variants does not include these overlapped variants
